# *Rhipicephalus appendiculatus* ticks transmit *Theileria parva* from persistently infected cattle in the absence of detectable parasitemia: implications for East Coast fever epidemiology

**DOI:** 10.1186/s13071-018-2727-6

**Published:** 2018-03-02

**Authors:** Cassandra L. Olds, Kathleen L. Mason, Glen A. Scoles

**Affiliations:** 1 Department of Veterinary Microbiology and Pathology, Washington State University, Pullman, WA USA; 2 USDA, ARS, Animal Disease Research Unit, Washington State University, Pullman, WA USA; 3 Present Address: Department of Entomology, Plant Pathology and Nematology, University of Idaho, Moscow, ID USA

**Keywords:** *Theileria parva*, *Rhipicephalus appendiculatus*, East Coast fever, Reservoir competence, Persistent transmission, Endemic stability

## Abstract

**Background:**

East Coast fever (ECF) is a devastating disease of cattle and a significant constraint to improvement of livestock production in sub-Saharan Africa. The protozoan parasite causing ECF, *Theileria parva*, undergoes obligate sexual stage development in its tick vector *Rhipicephalus appendiculatus*. Tick-borne acquisition and transmission occurs transstadially; larval and nymphal ticks acquire infection while feeding and transmit to cattle when they feed after molting to the next stage. Much of the current knowledge relating to tick-borne acquisition and transmission of *T. parva* has been derived from studies performed during acute infections where parasitemia is high. In contrast, tick-borne transmission during the low-level persistent infections characteristic of endemic transmission cycles is rarely studied.

**Methods:**

Cattle were infected with one of two stocks of *T. parva* (Muguga or Marikebuni). Four months post-infection when parasites were no longer detectable in peripheral blood by PCR, 500 *R. appendiculatus* nymphs were fed to repletion on each of the cattle. After they molted to the adult stage, 20 or 200 ticks, respectively, were fed on two naïve cattle for each of the parasite stocks. After adult ticks fed to repletion, cattle were tested for *T. parva* infection by nested PCR and dot blot hybridization.

**Results:**

Once they had molted to adults the ticks that had fed as nymphs on Muguga and Marikebuni infected cattle successfully transmitted *Theileria parva* to all naïve cattle, even though *T. parva* infection was not detectable by nested PCR on salivary gland genomic DNA of a sample of individual ticks. However, a salivary gland homogenate from a single Marikebuni infected tick was able to infect primary bovine lymphocytes. Infection was detected by nested p104 PCR in 3 of 4 calves and detected in all 4 calves by *T. parva* 18S nested PCR/dot blot hybridization.

**Conclusion:**

We show that *R. appendiculatus* ticks are able to acquire *T. parva* parasites from infected cattle even in the absence of detectable parasitemia. Although infection was undetectable in a sample of individual ticks, cumulatively as few as 20 ticks were able to transmit *T. parva* to naïve cattle. These results have important implications for our understanding of *T. parva* transmission by *R. appendiculatus* in ECF endemic regions.

## Background

Acute infection of cattle with *Theileria parva* causes a severe lympho-proliferative disease known as East Coast fever (ECF), one of the most significant tick-borne diseases of cattle in Africa [1–3]. *Theileria parva* most likely co-evolved with African buffalo (*Syncerus caffer*) as its vertebrate primary host, which display no symptoms of disease when infected [1, 3]. Transmission of *T. parva* to domestic cattle occurs during the feeding of the infected tick vector, *Rhipicephalus appendiculatus. Theileria parva* sporozoites are released, together with saliva, from tick salivary glands into the feeding site where they infect bovine lymphocytes and develop into the schizont stage, transforming the cells leading to a clonal expansion of schizont-infected lymphocytes. In some of the schizont infected lymphocytes the process of merogony occurs, resulting in the release of merozoites which invade erythrocytes, forming the tick infective piroplasm stage [1, 3–7]. The persistent infectious state is thought to be maintained through the slow continuous proliferation of schizont-infected cells, although events governing this process, or where the schizont infected cells reside are poorly understood [1].

The existence of a carrier state for *T. parva* was first described by Young et al. [8] in 1986, who stressed that persistent infection of cattle was likely “of great importance in maintenance of *T. parva* infection in the field”. Despite this, most of what is known about the tick-borne acquisition and transmission of *T. parva* by *R. appendiculatus* has been gathered through laboratory studies of cattle undergoing the short-lived acute stage of infection [9–14]. Animals that recover from acute disease can remain persistently infected and serve as reservoirs for *R. appendiculatus* ticks [15]. In areas where natural transmission occurs, a phenomenon known as endemic stability has been described as “a climax relationship between host, agent, vector and environment in which all coexist with the virtual absence of clinical disease” [1]. Under conditions of endemic stability only calves are seen to undergo acute infections and the resulting carrier state may last a number of years [1]. Recently the concept of endemic stability for *T. parva* has been broadened to include the concept that protection may be correlated with closely related avirulent *Theileria* species circulating naturally in the cattle population [16]. Endemic stability can now be seen as a product of the environment that also includes ecosystem services provided by the broader microbiome [16]. Even under this broader concept of endemic stability, it is important to understand the contribution to herd immunity made by persistent *T. parva* infections, whether naturally acquired or artificially induced through vaccination with live sporozoites.

Acute disease and mortality occur most frequently in areas where endemic stability is absent or has been disrupted [1, 17]. This could be due to the movement of naïve cattle from non-endemic to endemic zones, or the breakdown of tick control, which may have been reducing transmission pressures, limiting the opportunity for early infection of calves and subsequent development of a broad immune response. Endemic stability can also be disrupted by herd improvement programs that introduce more productive European cattle breeds, known to be more susceptible to infection, or by the introduction of virulent *T. parva* strains directly from African buffalo reservoirs at the interface between domestic cattle and wildlife [1, 17–19].

The only currently available method of immunization against acute disease caused by *T. parva* is the Infection and Treatment Method (ITM) of vaccination. Using ITM, cattle are infected with live, cryopreserved infectious sporozoites (which may be derived from multiple *T. parva* stocks), and immediately treated with long-acting tetracycline to attenuate the infection. This method of vaccination has been shown to result in life-long protection against acute disease, although breakthroughs have been reported to occur [20, 21]. The Muguga stock of *T. parva* is one component of the ITM cocktail [22], and as the most widely used laboratory stock of *T. parva* it has formed the basis for much of our understanding of immune responses to *T. parva* and ECF. It has been suggested that the Muguga stock does not induce a carrier state and is cleared from the body one to three months after infection. It has also been suggested that this stock is not tick transmissible after the acute stage of infection has passed [1, 23–26]; however, there is no way of knowing from these previous studies if the parasite is truly absent, or simply undetectable due to lower sensitivity of the assays available at the time. The Marikebuni laboratory stock [27, 28] is widely accepted as inducing a carrier state [23, 29] with circulating parasite DNA detectable in the cattle host for over 400 days after infection [24], remaining tick transmissible for seven months after inoculation [29]. In order to better understand the natural transmission dynamics that cattle might experience in the field we investigated the ability of *R. appendiculatus* ticks to transmit either the Muguga or the Marikebuni stock parasites to naïve cattle after feeding on persistently infected animals with no detectable parasitemia.

## Methods

### Tick and parasite stocks

*Rhipicephalus appendiculatus* ticks (Muguga colony) and *T. parva* stocks (Muguga and Marikebuni) were obtained from Dr. Ivan Morrison (Roslin Institute, Edinburgh, Scotland). The tick colony was originally collected in Kenya but had been maintained at the Roslin institute on cattle for ≈ 20 years. At the Animal Disease Research Unit (ADRU) ticks were reared on male Holstein calves and kept at 26 °C, 92% relative humidity (RH) and a 14.5/9.5 h light/dark photocycle for molting and hardening, then moved to 15 °C, 92.5% RH and a 14.5/9.5 h light/dark photocycle for longer term holding. Ticks infected with *T. parva* were maintained continuously at 26 °C, as above.

### Infection and treatment of calves with *T. parva* Muguga and Marikebuni

Two male Holstein calves 3 months-old were infected with cryopreserved *T. parva* sporozoites by needle inoculation above the left scapular lymph node and co-treated with 20 mg/kg long acting oxytetracycline (Bio-mycin 200, Boehringer Ingelheim, Ingelheim am Rhein, Germany). Calf C-1448 was infected with Muguga stabilate P2014/2 produced from the salivary glands of 10 ticks (10 tick equivalents), determined by quantitative p104 PCR [30] to contain 1 × 10^2^ *T. parva* sporozoites. Calf C-1445 was infected with 10 tick equivalents of Marikebuni stock ED64, determined by quantitative p104 PCR [30] to contain 3 × 10^4^ *T. parva* sporozoites. To track infection, daily rectal temperatures were recorded from the day of inoculation until day 21 post-infection. Daily blood samples were taken from the jugular vein on day 0 and on days 5–25 post-infection and thereafter at weekly intervals for the duration of the study.

### Monitoring *T. parva* infection of C-1445 and C-1448, DNA extraction and PCR

To monitor the presence of *T. parva* in C-1445 and C-1448, genomic DNA was extracted from 200 μl of whole blood and eluted into 50 μl of elution buffer. All DNA extractions were performed using the DNEasy Blood and Tissue kit (QIAGEN GmbH, Hilden, Germany). Unless otherwise stated, PCR reactions were carried out in 32 μl volumes using 2 μl of template genomic DNA, 1.0 μM forward primer, 1.0 μM reverse primer, 200 μM of dATP, dCTP, dGTP, dTTP, 1.3 U taq, 2.0 mM MgCl_2_ in 10× reaction buffer (FastStart PCR Polymerase dNTPack kit, Roche, Basel, Switzerland). For nested PCR reactions, the second round of PCR used 0.1 μl of the first PCR reaction together with 1.0 μM forward primer, 1.0 μM reverse primer, 200 μM of dATP, dCTP, dGTP, dTTP, 1.25 U taq, 2.0 mM MgCl_2_ in 10× reaction buffer and 1X RediLoad (Invitrogen, Carlsbad, USA). Reactions were set up in 8 well strips, with the 8th well of every strip serving as a no template control.

Successful DNA extraction was confirmed by PCR detection of genomic bovine DNA using bovine cytochrome *b* gene as a target. Cytb1 (5'-CCA TGA GGA CAA ATA TCA TTC TG-3') and Cytb3 (5'-GGG TGT TCD ACT GGY TGB CCY CC-3') primers with an annealing temperature of 55 °C amplified a 600 bp product [31]. *Theileria parva* DNA was detected using a nested PCR targeting the p104 gene [24, 26]. External primers IL3231 (5'-ATT TAA GGA ACC TGA CGT GAC TGC-3') and IL755 (5'-TAA GAT GCC GAC TAT TAA TGA CAC C-3') [24] were used with an annealing temperature of 60 °C, amplifying an 827 bp product. Internal forward p104 primer (5'-GGC CAA GGT CTC CTT CAG ATT ACG-3') and reverse primer (5'-TGG GTG TGT TTC CTC GTC ATC TGC-3') [26] were used with an annealing temperature of 55 °C to amplify an internal product of 277 bp.

All PCR reactions were run on a Bio Rad C1000 Touch^TM^ Thermal Cycler with an initial pre-melt step of 94 °C for 4 min 30 sec followed by 35 cycles of 94 °C for 30 sec, primer specific annealing temperature for 30 sec and extension at 72 °C for 30 seconds. A final extension step at 72 °C for 4 min 30 sec completed the reaction. PCR products were visualized by 2% agarose gel electrophoresis with 1:10,000 SYBR safe (Invitrogen, Carlsbad, USA).

### *Rhipicephalus appendiculatus* acquisition of *T. parva* from C-1445 and C-1448

*Rhipicephalus appendiculatus* acquisition of *T. parva* from C-1445 and C-1448 was attempted four months after infection. Five-hundred nymph ticks were applied in tick feeding patches secured to the back of each calf. During the tick-feeding period, daily jugular vein blood samples were drawn and whole blood samples were tested for the presence of *T. parva* DNA as described above. Ticks were allowed to feed to repletion after which they were collected from the feeding patch and kept in incubators to molt into adult stage ticks.

*Theileria parva* infection rates in the salivary glands of adult ticks, fed as nymphs on C-1445 and C-1448, were measured using nested p104 PCR. To stimulate sporozoite maturation in tick salivary glands 25 male and 25 female adult ticks were fed on the ears of rabbits for four days [32]. After feeding on rabbits, adult ticks were dissected and salivary glands from individual ticks were placed into 100 μl of RPMI 1640 media supplemented with 10% heat-inactivated fetal calf serum, 2 mM L-glutamine, 100 units/ml penicillin, 50 μg/ml streptomycin and 5×10^-5^ M β-mercaptoethanol (cRPMI). Salivary glands were homogenized with a sterile micropestle in a 1.5 ml microcentrifuge tube. Half the homogenate was used to infect bovine peripheral blood mononuclear cells (PBMC) and the remainder stored at -20 °C until genomic DNA was extracted and tested by nPCR for the presence of *T. parva* DNA.

### Infection of PBMC with *T. parva* from tick salivary gland homogenate

*Theileria parva* sporozoites from tick salivary glands are able to infect bovine PBMCs in vitro. To further evaluate the *T. parva* infection in adult ticks, 2 × 10^5^ PBMCs from an uninfected donor calf were combined with 50 μl of the tick salivary gland homogenate from each tick. The cells were incubated for one hour at 37 °C in 5% CO_2_. Samples were agitated every 15 minutes to resuspend cells. Following incubation, 1 ml of cRPMI was added to each tube and centrifuged at 500× *g* for 3 min to wash the cells. Pelleted cells were resupended in 100 μl of cRPMI and plated into individual wells of a 96 well plate. Cells were incubated for 3 weeks at 37 °C in 5% CO_2_ after which, cells were harvested and genomic DNA extracted. DNA was eluted into 30 μl of elution buffer and kept at -20 °C until use. Samples were tested for the presence *T. parva* DNA by nested p104 PCR as described above.

### DNA extraction from tick salivary glands and PCR detection of *T. parva*

Genomic DNA was extracted from the remaining 50 μl of tick salivary gland homogenate using the DNEasy Blood and Tissue kit protocol for extraction of DNA from tissue. DNA was eluted into 30 μl of elution buffer. To confirm that DNA was successfully isolated from each salivary gland sample, tick DNA was detected using primers 16S+1 (5'-CTG CTC AAT GAT TTT TTA AAT TGC TGT GG-3') and 16S-1 (5'-CCG GTC TGA ACT CAG ATC AAG T-3') targeting a 460 bp fragment of the tick mitochondrial 16S rRNA gene [33]. *Theileria parva* DNA was detected by nested p104 PCR in the same manner as described for bovine blood.

### Tick transmission of *T. parva* from persistently infected to naïve hosts

To determine if adult ticks fed as nymphs on persistently infected cattle were able to transmit *T. parva* to naïve cattle, ticks were fed on four naïve calves*.* On calf C-1470, 10 male and 10 female adult ticks from Marikebuni infected calf C-1445 were fed. 100 male and 100 female adult ticks from C-1445 were fed on calf C-1471. On calf C-1472, 10 male and 10 female adult ticks from Muguga infected calf C-1448 were fed while on calf C-1473, 100 male and 100 female adult ticks from C-1448 were fed. Ticks were applied as a group to each calf in a feeding patch and allowed to feed until female ticks detached at repletion. After all the female ticks had naturally detached male ticks were manually removed using forceps. Salivary glands were dissected from all males and replete females; DNA was extracted and tested for the presence of *T. parva* DNA by nested p104 PCR.

Animals were monitored daily for symptoms of ECF such as fever (rectal temperature above 39.5 °C), swollen lymph nodes, loss of appetite and coughing. Peripheral blood was sampled daily to confirm parasite transmission by PCR. Nested p104 PCR was carried out daily on genomic DNA extracted from both whole blood and separated lymphocytes. Because the schizont stage of *T. parva* proliferates in lymphocytes, PBMCs were isolated daily from 5 ml of blood using ficol-paque gradient centrifugation to increase the sensitivity of parasite detection. DNA was extracted from 5×10^6^ PBMCs and eluted into 50 ul of elution buffer. To increase the quantity of DNA tested for each animal, 10 reactions were performed for each lymphocyte sample. For each animal, days 0 and 5–21 post-tick application were tested. In addition to p104 PCR results, PCR targeting the *T. parva* 18S rRNA gene (described below) was performed on each sample, 18S rRNA PCR products were detected by Southern blotting; positive samples were sequenced for confirmation.

### PCR and Southern blot detection of *T. parva* targeting the 18S rRNA gene

Nested 18S rRNA PCR reactions were performed in triplicate for each sample to allow for sufficient sample material. First round reactions were carried out using forward primer *Theileria* 18S_F1 (5'-GAG GGA GCC TGA GAA ACG-3') and reverse primer *Theileria* 18S_R1 (5'-GGT ATC TGA TCG TCT TCG ATC C-3') with an annealing temperature of 65 °C. Second round PCR primers were Bab 18S_437-461 F (5'-AAT CCT GAC ACA GGG AGG TAG TGA C-3') and Bab 18S_898-873 R (5'-CTA AGA ATT TCA CCT CTG ACA GT-3') [34]. The products from three PCR reactions were pooled together and visualized on a Southern dot blot by hybridization with a digoxigenin (DIG)-labeled *T. parva* probe.

The *T. parva* probe was produced using forward and reverse primers each with a 5' DIG label and to increase the signal strength additional DIG labeled dUTPs added to the PCR mix during synthesis using the PCR DIG Probe Synthesis Kit (Roche, Basel, Switzerland). The template for PCR amplification of the probe was provided by the full length Muguga 18S rRNA gene cloned into pCR4 TOPO vector (Invitrogen, Carlsbad, USA). In a 50 μl probe synthesis reaction, 66 pg of template was added to 0.5 μM forward (5'-/5DigN/ GCT GCA TCG CTG GTC TCC-3'), and 0.5 μM reverse (5'-/5DigN/ CAT CCA GAC AAA GCG AAC TCC-3') primers, 200 μM dATP, dCTP, dGTP, 165 μM dTTP and 35 μM DIG-dUTP in 10× reaction buffer with a final concentration of 75 μM MgCl_2_. Probe synthesis reactions were run according to the manufacturer’s instructions. After PCR cycling was complete, the DIG-labeled probe was heated to 95 °C for 5 min and immediately transferred to ice after which, the product was added to 20 ml of DIG Easy Hyb buffer (Roche, Basel, Switzerland) pre-warmed to 42 °C. The probe was reused for each blot by storing at -20 °C between uses. Before each reuse, the probe was heated at 72 °C for 10 min. Positively charged Magnacharge nylon transfer membrane (GE Osmonics, Minnetonka, USA) was wet in 0.1% saline-sodium citrate (SSC) buffer (15 mM NaCl, 1.5 mM sodium citrate, pH 7). The membrane was placed onto a 96 well dot blotter, 35 μl of combined PCR products was mixed with 100 μl 0.1% SSC and heated to 95 °C for 2 min then immediately transferred to ice. Samples remained on ice until loading into individual wells of a 96 well dot blotter and passed through using vacuum suction. Membranes with the DNA fragments were cross-linked in a UV cross-linker (1200 μJoules × 100, UV Stratalinker 2400). Once cross-linked, membranes were sealed within hybridization bags (Roche, Basel, Switzerland) and blocked with 30 ml of DIG blocking buffer (DIG Wash and Block Buffer Set, Roche, Basel, Switzerland) for 30 min with gentle rocking. Following blocking, the membrane was equilibrated in DIG Easy Hyb buffer for 30 min. Hybridization with the probe was carried out at 42 °C for one hour with rocking. Following hybridization, the membrane was briefly washed in DIG Easy Hyb Wash buffer and then blocked with 30 ml Blocking buffer for 30 min. Following blocking, a 1:20,000 dilution of anti-Digoxigenin-AP Fab fragments (Roche, Basel, Switzerland) in DIG blocking buffer was added and incubated for 30 min at room temperature with rocking. The membrane is then washed twice in 100 ml DIG washing buffer for 15 min followed by equilibration in 5 ml of DIG Detection buffer. 20 μl of CDP-*Star* (Roche, Basel, Switzerland) was added to 2 ml DIG detection buffer, incubated for 5 min before being exposed to Blue Lite Autorad film (GeneMate, Radnor, Pennsylvania) for 10 s.

Where positive Southern blot dots were observed, the remaining PCR product was cleaned using the QIAquick PCR Purification Kit and sequenced using the Bab 18S_437-461 primer. Sequences were compared to the *T. parva* 18s rDNA sequences obtained from GenBank (HQ895984.1) using the Clustal Omega (https://www.ebi.ac.uk/Tools/msa/clustalo/) multiple sequence alignment tool.

## Results

### Infection of calves with *T. parva*

**Fig. 1:**
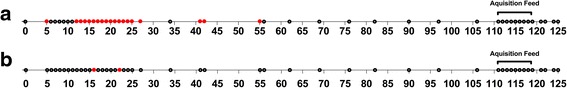
Detection of *T. parva* DNA by nested p104 PCR in Marikebuni infected calf C-1445 (**a**) and Muguga infected calf C-1448 (**b**). Dates tested post-infection are represented by circles; circles are shown in red on dates where parasite DNA was detected

Inoculation of calves C-1445 and C-1448 with *T. parva* sporozoites and concurrent treatment oxytetracycline resulted in mild clinical disease characterized by marked enlargement of the lymph node draining the site of sporozoite inoculation. At no point was fever (rectal temperature above 39.5 °C) observed and no additional oxytetracycline treatment was required. Parasites were detectable by nested p104 PCR in peripheral blood of the Marikebuni-infected calf C-1445 until day 55 post-infection and until day 22 in the Muguga-infected calf C-1448 (Fig. 1). Beyond this, parasites were not detectable in weekly blood samples from either animal, either before or during tick feeding at 4 months post-infection.

### Infection rates in adult ticks fed as nymphs on persistently infected cattle C-1445 and C-1448

**Fig. 2:**
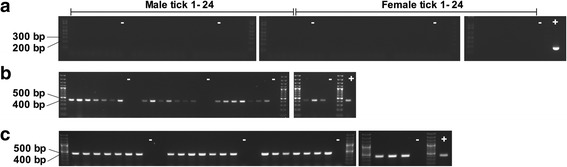
Determining *T. parva* infection rate by p104 nested PCR in adult ticks fed as nymphs on Marikebuni infected calf C-1445 (**a**). Tick 16S PCR confirms genomic DNA was successfully isolated from all male (**b**) and female (**c**) ticks

**Fig. 3:**
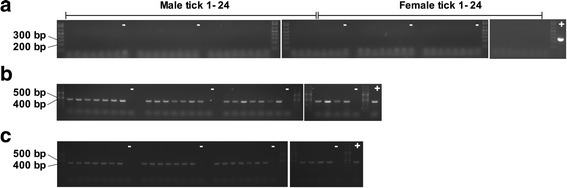
Determining *T. parva* infection rate by p104 nested PCR in adult ticks fed as nymphs on Muguga infected calf C-1448 (**a**). Tick 16S PCR confirms genomic DNA was successfully isolated from all male (**b**) and female (**c**) ticks

**Fig. 4:**
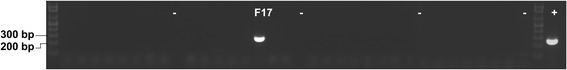
Detection of *T. parva* DNA by p104 nested PCR in lymphocyte cultures from Marikebuni-infected C-1445. *Theileria parva* DNA could only be detected in the lymphocyte culture from Female tick #17

After fed nymphs had molted to the adult stage, genomic DNA was successfully extracted from salivary gland homogenate of ticks fed on Marikebuni-infected calf C-1445 (Fig. 2b, c) and Muguga-infected calf C-1448 (Fig. 3b, c). No *T. parva* DNA could be detected in any of the 24 male or 24 female ticks analyzed (Figs. 2a and 3a). However, *T. parva* DNA was detected in lymphocytes incubated with Marikebuni female tick #17 salivary gland homogenate indicating viable sporozoites were present (Fig. 4).

### Transmission of *T. parva* to naïve calves by ticks fed on C-1445 and C-1448 four months post-infection

**Fig. 5:**
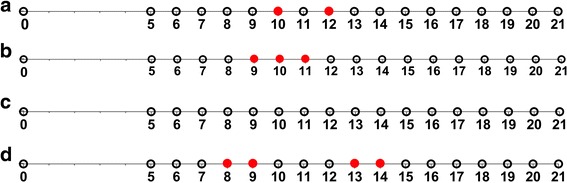
Detection of *T. parva* DNA by p104 nested PCR in 20 tick Marikebuni calf C-1470 (**a**), 200 tick Marikebuni calf C-1471 (**b**), 20 tick Muguga calf C-1472 (**c**) and 200 tick Muguga calf C-1473 (**d**). Dates tested post-infection are represented by circles; circles are shown in red on dates where parasite DNA was detected

**Fig. 6:**
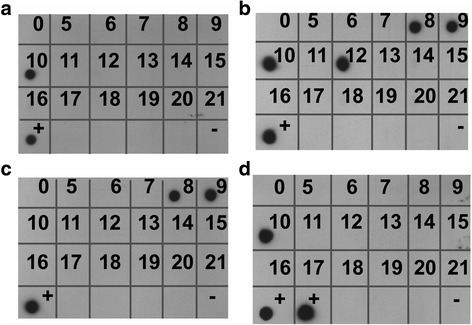
Detection of *T. parva* DNA by 18S rRNA gene PCR and Southern blotting in 20 tick Marikebuni calf C-1470 (**a**), 200 tick Marikebuni calf C-1471 (**b**), 20 tick Muguga calf C-1472 (**c**) and 200 tick Muguga calf C-1473 (**d**)

To determine if adult ticks that had been fed as nymphs on persistently infected cattle had been able acquire parasite levels sufficient to transmit *T. parva* to naïve calves, 20 (10 male and 10 female) or 200 (100 male and 100 female) ticks were fed, respectively on 2 different susceptible calves for each parasite stock. Adult ticks fed as nymphs on Marikebuni-infected C-1445 transmitted *T. parva* to naïve cattle C-1470 and C-1471 on which 20 and 200 ticks, respectively were applied. No parasites were detected in whole blood samples; however, using nested p104 PCR, *T. parva* DNA was detected in lymphocytes from C-1470 on day 10 and 12 (Fig. 5a) and on day 10 by 18S rRNA gene nPCR and Southern blotting (Fig. 6a). In C-1471 *T. parva* DNA was detected by p104 nPCR in lymphocytes on day 9, 10 and 11 (Fig. 5b) and on days 8, 9, 10 and 12 by 18S nPCR and Southern blotting (Fig. 6b).

**Fig. 7:**
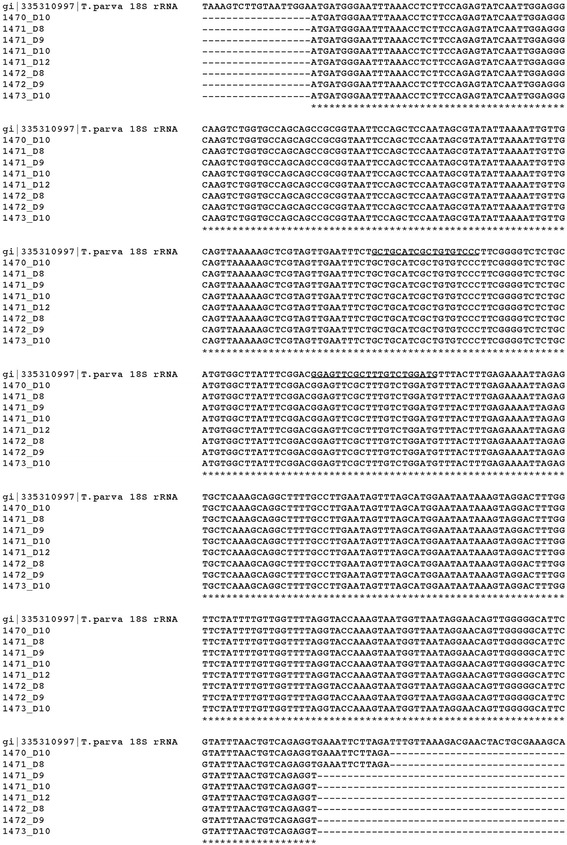
Multiple sequence alignment for samples that tested positive by Southern blot using a *T. parva* 18S rRNA gene reference sequence obtained from GenBank (accession # 335310997), priming sites for hybridization probe are underlined on the reference sequence

Ticks fed on Muguga-infected C-1448 transmitted *T. parva* to C-1472 and C-1473, on which 20 and 200 ticks, respectively were applied. *Theileria parva* DNA was detected in C-1473 lymphocytes on day 8, 9, 13 and 14 post-tick application using p104 nPCR (Fig. 5d) and on day 10 by 18S rRNA gene nPCR and Southern blotting (Fig. 6d). In C-1472, *T. parva* DNA was detected in lymphocytes by Southern blotting on day 8 and 9 (Fig. 6c) but was not detected using p104 nPCR (Fig. 5c). Sequences of the 18S rRNA gene PCR products for all animals were identified as *T. parva* (Fig. 7). No *T. parva* DNA could be detected in the salivary glands from replete ticks collected after transmission feeding on each calf using either p104 or 18S rRNA nPCR.

## Discussion

Our current understanding of tick-borne acquisition and transmission of *T. parva* has been derived primarily from studies of tick-pathogen-host interactions occurring during the acute stage of infection in animals undergoing severe ECF. During acute infection, high parasitemia is observed in the bovine host, which translates to quantifiable infection levels in ticks feeding on infected animals. Under natural field conditions, animals undergoing acute disease without treatment may not survive long enough for tick feeding to be completed making them dead-end hosts. Animals that do recover from acute stage infection may remain low-level carriers for many months. Acquisition and transmission of *T. parva* from these persistently infected cattle is thus more representative of the natural transmission cycle in areas where *T. parva* is endemic.

We have shown that even though parasites are undetectable in the peripheral blood at the time of tick feeding, ticks can and do acquire the parasite after feeding on cattle suggesting that red blood cells infected with the piroplasm stage are present. It is not known where or if *T. parva* sequesters in the bovine host, this study shows only that cattle remain competent reservoirs of *T. parva* and can infect ticks, leading to transmission, even after circulating parasitemia levels are below detection. This work confirms that these *T. parva* parasite stocks are tick transmissible for at least four months after infection. We have shown with this work that infections in ticks do not have to be high or detectable by currently available detection methods to transmit parasites to naïve hosts. Furthermore, transmission by ticks from these low level persistent infections resulted in sub-clinical infections, which required no treatment intervention. It follows from these results that cattle in the field that have been previously exposed to *T. parva* could serve as a source of infection for ticks, even if circulating parasites in the blood cannot be detected. Likewise, ticks feeding on cattle in endemic regions may be infective even if parasites are at undetectable levels.

Published data derived from tick feedings during acute infections suggests that the infection level in ticks is related to the parasitemia of the infected bovine host on which it feeds [35]. The sensitivity of currently available assays is not high enough to confirm that the same relationship exists in ticks fed on persistently infected cattle. Successful transmission of parasites by tick populations where parasites cannot be detected has been described for *Babesia bovis* [36] but has not been described for *T. parva* until now*.* We have shown here that, although individual ticks fed on persistently infected hosts have undetectable levels of infection in their salivary glands, cumulatively (in groups of 20 or more) they were able to transmit parasites to susceptible bovine hosts. Although this transmission was demonstrated in a relatively small number of cattle, it was observed in all four cattle tested (2 for each parasite stock), it is likely that this observation would occur in a similar fashion on a larger scale.

The ability of ticks with unapparent infection levels to transmit *T. parva* has far reaching implications for field surveys determining *T. parva* infection rates in both ticks and cattle. Field infection surveys of endemic areas report overall infection rates in ticks below 5% with a low level of infection in individual ticks [8, 37–39]; however, in light of the results of this study, we suggest that this grossly underestimates the number of ticks infected with *T. parva.* Likewise, single blood samples taken from cattle in *T. parva* endemic regions during infection surveys may underestimate infection prevalence.

Because parasite numbers associated with persistently infected cattle are at levels very close to the detection thresholds on all of our assays we investigated ways to increase the sensitivity in both the tick vector and bovine host. For detection of low-level infections in tick salivary glands we incubated salivary gland homogenate with primary lymphocytes. Using this method we detected one infected tick where nested p104 PCR failed to detect any (Fig. 4). This method may be useful for investigating low-level tick infection rates in field collected ticks. As the primary site of replication for *T. parva* in the bovine host occurs in lymphocytes, we extracted lymphocyte genomic DNA to increase detection of infection in blood. We further increased sensitivity for the p104 nested PCR by running 10 replicates for each test day, effectively increasing the volume of template tested, increasing the sensitivity by one order of magnitude. Finally, for additional confirmation of transmission, we used dot blot hybridization with chemo-luminescent detection to increase the sensitivity of our 18S rRNA nPCR. Although more labor-intensive, these methods may also be employed in field surveys of *T. parva* prevalence in cattle.

Tick-based transmission models using persistently infected cattle as a source of infection may more accurately simulate the natural transmission pressures experienced by cattle in the field. Consequently, the study of transmission from persistently infected cattle may lead to a better understanding of the epidemiology of *T. parva* in endemic areas. Such models may also be useful as challenge models for studies testing the efficacy of future subunit vaccines [40]. In addition, our knowledge of *T. parva* immunobiology is strongly based on data collected from needle inoculation of host cattle. Needle inoculation excludes the potential impact of the tick on the route and quantity of infective pathogens introduced, eliminating the potential modulatory effects of tick salivary gland molecules on enhanced transmission and disease progression [41]. Using tick-based challenge models, infectious sporozoites are transmitted to cattle over several days and in the context of tick saliva [40, 42]. This is in sharp contrast to a one-time needle inoculation using a large number of cryopreserved sporozoites. Under conditions of endemic stability, a robust protective immune response is developed upon initial tick-borne exposure even in the absence of clinical disease. It is possible that exposure to low levels of naturally presented *T. parva* sporozoites allows the immune response to develop and act correctly, avoiding the immune dysregulation associated with severe disease [43]. Evaluation of the immune response to sporozoites naturally presented in the context of tick saliva is important to broaden our understanding of *T. parva* immunology, which has so far been founded solely on artificial needle challenge experiments.

In the broader context, a recent study observed an 89% reduction in mortality associated with *T. parva* infection in calves co-infected with less pathogenic *Theileria* species such as *Theileria mutans* and *Theileria velifera* [16]. Strikingly, co-infection with *T. mutans* and *T. parva* is identified to be advantageous to host growth rate compared to *T. parva* alone [44]. This suggests that the conventional concept of endemic stability may need to be expanded to include the broader community of tick-transmissible parasites [16] and uncovering the mechanism of this protection is essential. The advantage of co-infection with multiple strains of the same apicomplexan parasite is seen in malaria where infection of children with multiple *Plasmodium falciparum* genotypes is an indicator of lowered risk of clinical disease compared to single genotype infections [45], although once again, the mechanism remains unknown.

## Conclusion

We have shown that the levels of *T. parva* parasites circulating in the blood stream that are required for transmission by *R. appendiculatus* is very low, highlighting the co-evolution of this pathogen and tick vector. We suggest utilizing a laboratory model of persistent infection as a tool to simulate and investigate host-pathogen-vector interactions occurring in endemic regions.

## Abbreviations

ECF: East coast fever
ITM: Infection and treatment method
PBMC: Peripheral blood mononuclear cells
